# Aromatase Expression in the Hippocampus of AD Patients and 5xFAD Mice

**DOI:** 10.1155/2016/9802086

**Published:** 2016-05-19

**Authors:** Janine Prange-Kiel, Danuta A. Dudzinski, Felicitas Pröls, Markus Glatzel, Jakob Matschke, Gabriele M. Rune

**Affiliations:** ^1^Department of Cell Biology, University of Texas Southwestern Medical Center, 5323 Harry Hines Boulevard, Dallas, TX 75390, USA; ^2^Institute of Neuroanatomy, University Medical Center Hamburg Eppendorf, Martinistraße 52, 20246 Hamburg, Germany; ^3^Institute of Neuropathology, University Medical Center Hamburg Eppendorf, Martinistraße 52, 20246 Hamburg, Germany

## Abstract

Numerous studies show that 17*β*-estradiol (E_2_) protects against Alzheimer's disease (AD) induced neurodegeneration. The E_2_-synthesizing enzyme aromatase is expressed in healthy hippocampi, but although the hippocampus is severely affected in AD, little is known about the expression of hippocampal aromatase in AD. To better understand the role of hippocampal aromatase in AD, we studied its expression in postmortem material from patients with AD and in a mouse model for AD (5xFAD mice). In human hippocampi, aromatase-immunoreactivity was observed in the vast majority of principal neurons and signal quantification revealed higher expression of aromatase protein in AD patients compared to age- and sex-matched controls. The tissue-specific first exons of aromatase I.f, PII, I.3, and I.6 were detected in hippocampi of controls and AD patients by RT-PCR. In contrast, 3-month-old, female 5xFAD mice showed lower expression of aromatase mRNA and protein (measured by qRT-PCR and semiquantitative immunohistochemistry) than WT controls; no such differences were observed in male mice. Our findings stress the importance of hippocampal aromatase expression in neurodegenerative diseases.

## 1. Introduction

The synthesis of the steroid hormone 17*β*-estradiol (E_2_) is catalyzed by the enzyme aromatase, a member of the cytochrome P450 superfamily. Aromatase is not only expressed in the gonads of humans and rodents but also in their brains. In the human brain, aromatase mRNA, aromatase protein, and aromatase activity have been detected in the hippocampus, the amygdala, the preoptic area, the hypothalamus, and the neocortex [1–3]. Initially, the function of brain-derived E_2_ was thought to be restricted to the regulation of reproductive behavior and neuroendocrine processes in the hypothalamus (for review [2]). More recent studies, however, have revealed that locally produced E_2_ also protects the brain against a variety of neurological and neurodegenerative disorders, including Alzheimer's disease (AD) [4, 5].

Patients diagnosed with AD progressively lose cognitive function and memory. On the cellular level, the disease is characterized by a decrease in synaptic proteins and the loss of synapses and neurons, all of which primarily occur in the hippocampus, the cerebral cortex, and some subcortical brain regions [6–8]. One particular feature of AD is the enhanced production and aggregation of the neurotoxic amyloid beta peptide (A*β*) into oligomers and plaques, which induce the loss of synapses and neurons [9].

The neuroprotective function of brain-derived E_2_ in AD has been described in a transgenic mouse model for AD (APP23 mice), in which the animals develop A*β* plaques and other pathological changes observable in the brains of AD patients [10, 11]. APP23 mice were crossbred with aromatase-KO (Ar^−/−^) mice to test the influence of E_2_ on the formation of A*β* plaques. The resulting female progeny, which is Ar^+/−^ and therefore E_2_-haploinsufficient, demonstrated faster and more severe A*β* plaque formation and a less effective A*β* clearance compared to aromatase-expressing APP23 mice [12]. Ovariectomy of APP23 females, thus the elimination of their major source of systemic E_2_, did not mimic the effects of a genetically induced aromatase deficiency, which affected all aromatase-expressing tissues, including the brain. These results suggest that the brain-derived E_2_, rather than the ovary-derived E_2_, counteracts A*β* plaque formation and is therefore neuroprotective in female mice. Surprisingly, in male APP23/Ar^+/−^ mice, A*β* plaque production is reduced compared to APP23/Ar^+/+^ animals, indicating that the neuroprotective role of brain-derived E_2_ may be sex-dependent [13]. Sex differences can also be observed with respect to the prevalence and severity of AD in humans. Women have a higher risk of developing AD better than age-matched men, and the cognitive deterioration is faster and more pronounced in women than in men (for review [14]).

Although aromatase expression in the brain and the resulting local E_2_ synthesis have been shown to be important factors in protection against AD, very little is known about how AD influences the expression of aromatase in the human hippocampus, the brain region that is among the first to be affected by AD. In general, the expression of aromatase mRNA is regulated through the alternative use of multiple, promoter-specific first exons (for review [15]). These first exons, which remain untranslated, are spliced onto the coding exons 2 through 10 of the aromatase gene, resulting in numerous aromatase transcripts, all of which code for the same protein. Although alternative first exons are considered to be tissue-specific, very often multiple transcript variants are present in one tissue. In the human brain, for example, exon I.f is considered to be the predominant variant [16]. However, PII, I.3, and I.3T (all ovary-specific), and I.4 (adipose tissue-specific) can also be detected in the human brain [17, 18]. As each promoter contains at least one distinct regulatory element, the regulation of aromatase expression in the brain is extremely complex. Hardly anything is known on whether neurological diseases, such as AD, may influence the expression of aromatase and its various promoter-specific transcripts.

Using RT-PCR and immunohistochemistry, we compared the expression of aromatase mRNA and protein in postmortem hippocampal tissue of individuals diagnosed with AD and of individuals that did not have any neurodegenerative disease. We chose to analyze the CA4 region of the hippocampus because this region retains morphological integrity throughout the progress of AD better than, for example, CA1, where neuronal loss already occurs in patients with mild symptoms of AD [19]. We extended our analyses of aromatase expression to a mouse model for AD, the 5xFAD mice [20]. The neuronal tissue of these mice overexpresses a mutant form of the human amyloid precursor protein (APP), which bears three mutations known to cause familial AD (FAD). Additionally, a form of human presenilin 1 harboring two FAD mutations is overexpressed in the brain of these mice. 5xFAD mice show AD-like pathology as early as two months of age and develop cognitive defects at six months of age [20]. This animal model allowed us not only to study aromatase immunoreactivity but also to investigate the expression of aromatase mRNA and its promoter-specific variants in the hippocampi in a semiquantitative approach using real-time RT-PCR.

## 2. Methods

### 2.1. Tissue

#### 2.1.1. Postmortem Human Tissue (cf. Table S1 in Supplementary Material available online at http://dx.doi.org/10.1155/2016/9802086)

Brain tissue was obtained from autopsies routinely performed at the Institute of Neuropathology, University Medical Center Hamburg-Eppendorf, Germany. Alzheimer's disease was clinically and neuropathologically confirmed by applying current diagnostic standards. The use of specimens was in agreement with the regulations and ethical standards at the contributing hospitals. Hippocampi were dissected from coronal sections at the level of the lateral geniculate body and processed for paraffin embedding or snap-frozen in liquid nitrogen and stored at −80°C.

#### 2.1.2. 5xFAD Mice

5xFAD mice and WT control animals (C57BL/6J) were housed in the Animal Resource Center at the University of Texas Southwestern Medical Center (Dallas, TX, USA). The mice were kept under controlled conditions with a 12 h/12 h dark/light cycle and water and food available ad libitum. Male and female animals at the ages of 3 and 12 months were used for the study. The systemic hormone level of the younger female mice varies due to their estrous cycle. Therefore, the ovarian cycle of the 3-month-old females was monitored by analyzing their vaginal smear over at least four cycles. These females were sacrificed in the morning of proestrus to minimize variation in systemic hormone concentration. All 12-month-old female mice used in this study were acyclic as confirmed by analysis of vaginal smear over ten days. For tissue collection for immunohistochemistry, the mice were deeply anesthetized with a ketamine/xylazine/acepromazine cocktail and transcardially perfused with 4% PFA. The brains were dissected out, cryopreserved in isopentane, which was cooled with liquid nitrogen, and stored at −80°C. For RNA isolation, deeply anesthetized mice were decapitated; subsequently, the brains were quickly removed from the skulls and the hippocampi were dissected bilaterally. The hippocampal tissue was snap-frozen in liquid nitrogen and stored at −80°C until further usage.

### 2.2. Immunostaining

#### 2.2.1. Human Hippocampal Sections

For immunostaining analyses, paraffin-embedded hippocampi from donors diagnosed with AD (mean age 77.7 ± 39.5, range 54–92) were compared to hippocampi from patients without any known neurological disease (controls, mean age 77.2 ± 38.3, range 54–91). Samples of ten AD patients and ten neurological healthy donors were paired based on the patients' sex and age (compare Table S1, supplement, sample numbers 1–10).

Sections (4 *μ*m thick) of paraffin-embedded hippocampi were mounted on glass slides and dried overnight at 37°C. Subsequently, the sections were deparaffinized and gradually rehydrated by washing in XEM-200 (xylene substitute, Vogel, Giessen, Germany), ethanol (100%, 96%, 80%, and 70%), and distilled water. For antigen retrieval, the sections were microwaved in citrate buffer (9 mL 0.1 M citric acid and 41 mL 0.1 M sodium citrate in 500 mL H_2_O) for 2 × 13 min at 700 W. The sections were then incubated overnight at 4°C with antibodies against human aromatase (SM2222P, Acris, Herford, Germany, 1 : 100), microtubule-associated protein 2 (MAP2, AB5622, Millipore, Merck Chemicals, Schwalbach, Germany, 1 : 1000), or glial fibrillary acidic protein (GFAP, AB5804, Millipore, 1 : 1000), all of which were subsequently visualized with appropriate secondary antibodies, conjugated with Cy3 or Alexa Fluor 488 (Dianova, Hamburg, Germany, 1 : 750, 60 min, RT). Additionally, immunohistochemical analyses based on DAB precipitation were performed according to standard protocol using an HRP-conjugated secondary antibody (Vectastain, Linaris, Dossenheim, Germany). For image acquisition, a microscope (Axioskop 2) equipped with a digital camera (AxioCam) and imaging software (Axiovision 3.1, all Zeiss, Jena, Germany) was used.

#### 2.2.2. Mouse Hippocampal Tissue

Coronary sections of the brain (12 *μ*m thick) were cut through the hippocampus using a cryomicrotome (Leica, Buffalo Grove, IL, USA). The sections were mounted on glass slides and dried at RT for 1 hr. After blocking with normal goat serum (Jackson ImmunoResearch, West Grove, PA, USA, 3% in PBS, 30 min, RT), the sections were incubated with a polyclonal aromatase antibody (1 : 1000), which had kindly been provided by Dr. I. Azcoitia (Madrid, Spain) and had been characterized elsewhere [21]. Subsequently, the sections were incubated with a Cy3-labelled secondary antibody (goat-anti-rabbit, Jackson ImmunoResearch, 1 : 750, 1 h, RT) and the nuclei were counterstained with DAPI (Sigma-Aldrich, St. Louis, MO, USA).

#### 2.2.3. Image Analysis

All samples that were going to be compared within one analysis were processed at the same time and under identical conditions. All measurements were performed with the experimenter blinded with regard to condition/genotype of the specimen under investigation. Confocal images of immunostained tissue sections were taken with an LSM 510 Meta Confocal Microscope (Zeiss, Germany, for human tissue) or with a TCS SP5 (Leica, USA, for mouse tissue) at the level of the section at which the staining was strongest as determined per visual inspection by the experimenter. The images were further analyzed with the help of a cell imaging system (Openlab 2.2.5, Improvision, Coventry, UK).

For every human hippocampus, a total of 30 images of the CA4 region were acquired (five images each from six sections). Conditions for image acquisition were kept constant for the matched pairs. For image analysis, first a threshold value was determined by using staining controls in which the primary antibody had been omitted. Only pixels with an intensity higher than this threshold value were considered in the subsequent calculation of the relative staining index. The relative staining index was calculated as previously described [22, 23]. The number of pixels and the intensity of each pixel, expressed as a value between 0 and 255 on a grey scale, were measured in an area of defined size in the perinuclear cytoplasm of five pyramidal neurons on every image. To avoid bias, the five uppermost and leftmost pyramidal neurons on each picture were used for the analysis. The relative staining index was determined by multiplying the amount of pixels with these pixels' individual signal intensity. The resulting value for the control tissue was set as 1 and the value for the AD tissue was set in relation to this control value. Comparison of the relative staining indices for aromatase was performed for each age- and sex-matched pair using the dependent *t*-test for matched samples. A *p* value of *p* ≤ 0.05 was considered to indicate a statistically significant difference.

For the mouse tissue, 30 images per animal were taken from both the CA1 and the CA3 region of the hippocampus (3 images each from 10 sections). Again, all parameters for image acquisition remained constant for all specimens. Due to the smaller size of the mouse hippocampus, signal measurement was performed slightly different in the mouse than in the human. Four squares of a defined size were laid over the pyramidal cell layer in each image and the image area defined by these squares was subsequently analyzed. Like in the human tissue, after determining the background threshold, the imaging software calculated the signal intensity of this area. The relative staining index was determined as described for the human tissue (i.e., multiplying the number of pixels by the intensity of the pixels). Subsequently, the average relative staining index was calculated for each animal and these values were used to determine the group average. ANOVA was used to analyze differences in the relative staining index among groups. Significant ANOVA was followed by a post hoc test (LSD), and *p* values ≤ 0.05 were considered to be significant.

### 2.3. PCR

#### 2.3.1. RT-PCR in Human Tissue

Cryopreserved hippocampal tissue of postmortem human brains was used to analyze which tissue-specific first exons of aromatase are expressed in healthy and AD-affected hippocampal tissue. A total of eight samples was used: 4 samples were obtained from brains of patients without neurological diseases and 4 samples from brains of patients diagnosed with AD (compare Table S1, supplement, sample numbers 11–18). Total RNA was isolated from 60 mg hippocampal tissue using the RNeasy Kit (Qiagen, Hilden, Germany) according to the standard protocol provided by the manufacturer. Following DNase treatment (Qiagen), aromatase cDNA was synthesized in a reverse transcription reaction using Phusion polymerase (Finnzyme Biolabs, Fisher Scientific, Schwerte, Germany) and an aromatase-specific primer (5′-TCT AGT GTT CCA GAC ACC TGT CTG AG-3′). Each PCR mix (50 *μ*L) contained 10 mM Tris-HCl, 50 mM KCl, 2 mM MgCl_2_, 0.1% Triton, 200 *μ*M of each dNTP, 500 mM primers (forward and reverse, cf. Table S2), 1 U Phusion polymerase, and 10 *μ*L cDNA. After initial denaturation (30 s, 98°C), the PCRs were carried out for 35 cycles; each cycle consisted of 10 s denaturation (95°C), 20 s annealing at a primer-specific temperature (cf. Table S2), and 2 min extension (72°C). A final extension step was performed at the end of the run (72°C, 10 min). Each PCR run included reactions without cDNA to control for potential external contaminations. The PCR products were electrophoresed on a 1% agarose gel, stained with ethidium bromide, and visualized under UV light. The following first exons were studied: PII (ovary-specific), I.f (brain-specific), I.3 (ovary-specific) and I.4 (specific for adipose tissue), and I.6 (bone-specific). The used primers had previously been described by Yague et al. [18]. All primer pairs spanned between the tissue-specific first exon and exon II; the lengths of the resulting PCR products were calculated based on exon sequences published by Cai et al. [24], Honda et al. [16], Mahendroo et al. [25], Shozu et al. [26], and Simpson et al. [27].

#### 2.3.2. Real-Time RT-PCR in Mouse Tissue

For total RNA isolation with trizol (LifeTechnologies, Grand Island, NY, USA) the two hippocampi from each animal were combined. After treatment with DNase (Life Technologies, USA), total RNA quantity and quality of all samples were measured with a UV spectrometer (NanoDrop, ThermoScientific, Pittsburgh, PA, USA). Only samples with an A260/A280 ratio > 1.9 were included in the experiments. Additionally, RNA integrity was confirmed by analyzing random samples with a bioanalyzer and the appropriate RNA chip (Agilent Technologies, Santa Clara, CA, USA) by the Genomic and Microarray Core Facility at UT Southwestern. Total RNA (1 *μ*g) was reverse-transcribed, using the SuperScript VILO cDNA synthesis kit (Life Technologies, USA). Real-time PCR was performed on a CFX348 Real-Time PCR Detection System (BioRad, Hercules, CA, USA) in 384-well plates. Each sample was run in triplicate with each 10 *μ*L-reaction consisting of 1.3 *μ*L cDNA (prediluted 1 : 10), 5 *μ*L Power SYBR Green PCR Master Mix (Life Technologies, USA), 1.2 *μ*L primer mix (forward and reverse, final concentration of each primer: 0.3 *μ*M, IDT, Coralville, IA, USA), and 2.5 *μ*L H_2_O. The real-time PCR conditions were 95°C for 10 min (initial activation) followed by 40 cycles of 95°C, 15 s (denaturation), and 60°C for 1 min (annealing/extension). At the end of the run, a melt curve was generated. Relative gene expression was calculated using the comparative cycle threshold (ΔΔCt) method, using actin beta mRNA as the reference gene. The data were analyzed with *t*-tests, *p* values < 0.05 indicating significant differences. Primer sets (cf. Table S3) for actin beta, aromatase total, and ovarian aromatase were designed based on their published mRNA sequences (GI: 145966868 - actin beta; GI: 1402594 - aro_ovary; GI: 156139071 - aro_total) using Primer Express 3.0 software (Life Technologies, USA). The primers for the ovary-specific exon (aro_ovary) span portions of this exon; the primers for the aromatase total (aro_total) hybridize to exons 8 and 9. The primers for the detection of the brain-specific first exon had been published by Yilmaz et al. and span the junction between exons I and II [28]. All primer sets produced amplicons of the expected size and sequence.

## 3. Results

### 3.1. Human Hippocampal Neurons Expressed Aromatase

**Figure 1:**
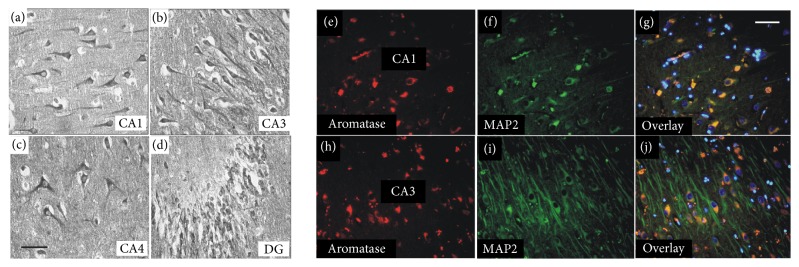
*Neurons in all regions of the human hippocampus expressed aromatase* (tissue obtained from an 89-year-old woman without any known neurological disease). (a–d) Immunohistochemical staining (DAB) for aromatase resulted in a strong signal in all hippocampal regions. (e–j) Coimmunostaining for aromatase (e, h) and the neuronal marker MAP2 (f, i) demonstrated that primarily hippocampal neurons expressed aromatase. Aromatase-expressing neurons are orange in the overlay, and nuclei were stained blue with DAPI (g, j). Images were taken from the CA1 region (e–g) and the CA3 region (h–j). Scale bar: 50 *μ*m; CA: cornu ammonis; DG: dentate gyrus.

As a first step, the distribution of aromatase protein in the human hippocampus was studied. Immunohistochemical staining of hippocampal sections from postmortem human brain tissue with an aromatase antibody revealed that it is primarily cells with a neuron-like morphology that express aromatase. This staining pattern was observed in all regions of the hippocampus, namely, all regions of the cornu ammonis (CA1, CA3, and CA4) and in the dentate gyrus (DG) (Figures 1(a)–1(d)). Costaining for aromatase and the neuronal marker MAP2 confirmed that most of the aromatase-positive cells were neurons (Figures 1(e)–1(j)). Based on the location and size of the neurons, it was concluded that the aromatase-positive neurons in the cornu ammonis and DG were pyramidal cells and granule cells, respectively. At the subcellular level, aromatase was detectable in somata, dendrites, and axons, with particularly strong immunoreactivity in the somata and in the initial segments of the neurites. Only a small portion of the aromatase-positive cells did not show any immunoreactivity for MAP2, indicating that a few nonneuronal cells also express aromatase. Costaining of aromatase and the astrocyte marker glial fibrillary acidic protein (GFAP) identified these cells as astrocytes. However, the majority of GFAP positive cells was aromatase-negative (Figure S1).

### 3.2. Aromatase Expression Was Higher in the Hippocampi of AD Patients

**Figure 2:**
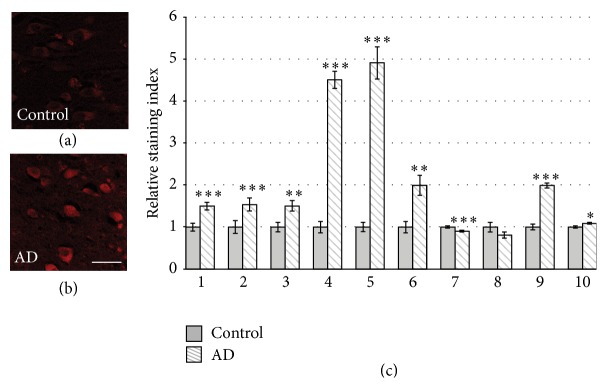
*Aromatase expression was higher in the hippocampi of brains from patients with AD than in control brains*. (a, b) Immunohistochemical staining in the CA4 region of postmortem hippocampi from a patient diagnosed with AD (b) and a sex- and age-matched control (a) (scale bar: 20 *μ*m). (c) Semiquantitative analysis of aromatase immunoreactivity, expressed as the relative staining index, demonstrated that in most cases aromatase reactivity was higher in the hippocampi obtained from AD brains than in the sex- and age-matched control hippocampi. The numbers for each matched pair refer to Table S1 (sample numbers 1–10) (^*∗*^ *p* ≤ 0.05, ^*∗∗*^ *p* ≤ 0.01, ^*∗∗∗*^ *p* ≤ 0.001; bars represent mean ± SEM).

Next, to identify potential differences in hippocampal aromatase expression between brains from patients with and without AD, immunofluorescent staining for aromatase of neurons was analyzed in sex- and age-matched pairs of postmortem tissue (Figures 2(a) and 2(b)). We had to restrict the analysis to the CA4 region of the hippocampus because neuronal loss due to AD in CA1 and CA3 made a thorough analysis of these regions impossible. This AD-induced neuronal loss, in particular in CA1, has been described before by others [19]. In eight out of ten pairs, the immunoreactivity was significantly higher in the brain with AD pathology than in the control brain. Only one pair displayed a reverse result and another pair did not show any significant difference (Figure 2(c)).

### 3.3. Various Aromatase Promoters Were Expressed in the Human Hippocampus

**Figure 3:**
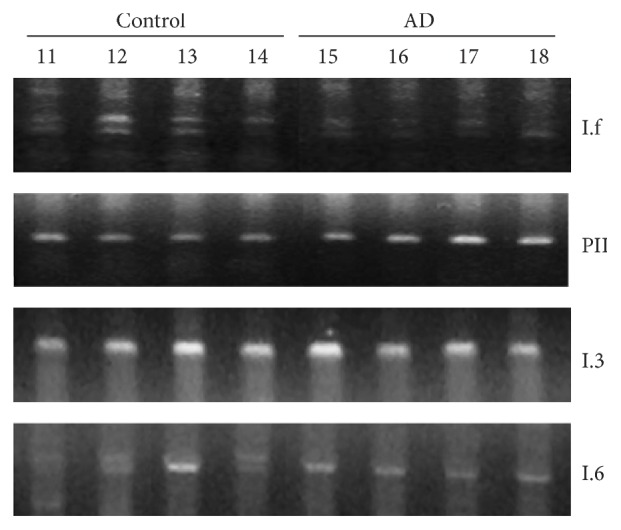
*Various types of aromatase first exons were expressed in the hippocampi from patients without neurological diseases and patients with AD*. RT-PCR using primer sets specific for different first exons of aromatase mRNA showed that several different promoters, namely, I.f, PII, I.3, and I.6, regulate aromatase expression in the hippocampus. The respective first exons were expressed in healthy hippocampi as well as in hippocampi from patients with AD. The numbers given for each sample refer to Table S1 (sample numbers 11–18).

In order to better understand potential mechanisms of the regulation of aromatase expression, promoter usage in hippocampal tissue of brains with and without AD pathology was studied by RT-PCR (Figure 3). Primers that target the first exons I.f, PII, I.3, I.4, and I.6 of the aromatase transcript were used for this set of experiments, as those first exons previously have been described to be expressed in the brain [17, 18]. PCR products of the expected size were detectable in hippocampal tissue from AD patients and control patients using the primers for I.f, PII, I.3, and I.6. The size of the band that was observed with the primer set for exon I.6 (approx. 910 bp) indicated that a splice variant that contains exons I.6 and I.3, as well as the interjacent intron (variant H2) [26], was expressed in hippocampi of both AD patients and control patients. However, no band of the expected specific size for exon I.6 (164 bp) could be detected; nor did the use of a primer pair against exon I.4 result in a specific PCR product.

### 3.4. Aromatase mRNA Expression as well as Protein Expression Was Sex-Specific in 5xFAD Mice

Although human hippocampal tissue provides important information on the expression of aromatase in AD, it does not allow studying potential changes in aromatase expression over the course of the disease. To overcome this problem, analysis of aromatase expression was extended to a mouse model for AD, the 5xFAD mouse, which shows AD-like pathology already at an early age. The total amount of aromatase mRNA expressed in the hippocampi of these mice was detected by a primer pair that spans parts of exons 8 and 9 and therefore detects all variants of aromatase mRNA. Similar to humans, in mice, several variants of aromatase mRNA exist, which are under the control of tissue-specific promoters and can be distinguished by specific first exons. Here, primers directed against the first exons were used to analyze the brain- and ovary-specific variants (Table S3) and thereby to further break down potential differences in the expression of total aromatase mRNA.

**Figure 4:**
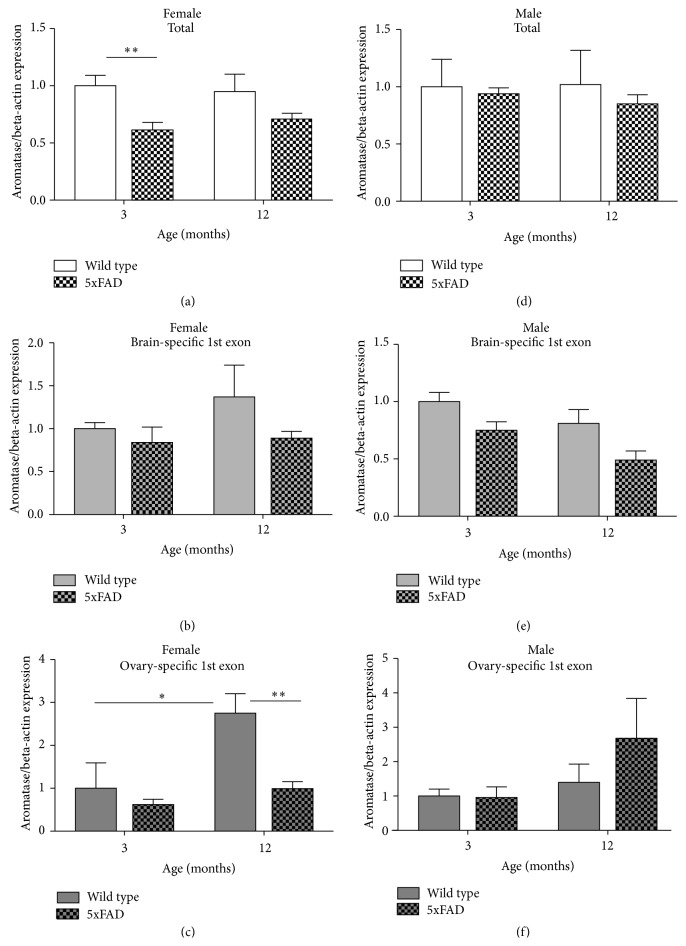
*Aromatase mRNA expression differed in hippocampi of female and male 5xFAD mice and first exon-specific aromatase expression changed with age*. In the hippocampi of female mice, the total aromatase expression was lower in the 5xFAD mice, with a significant difference in the 3-month-old animals (a), whereas no such difference was detectable in male animals (d). Expression of the tissue-specific first exons did not differ significantly in the male hippocampi, neither between the genotypes nor over time (e, f). In female mice, the expression of the ovary-specific first exon was significantly higher in the 12-month-old WT animals compared to the 3-month-old WT animals and the 12-month-old 5xFAD mice (c). However, expression of the brain-specific first exon did not differ significantly in the female mice (b) (^*∗*^ *p* ≤ 0.05, ^*∗∗*^ *p* ≤ 0.01; bars represent mean ± SEM, *n* = 6).

Interestingly, sex-specific differences in the expression pattern of aromatase were detected. In males, no significant differences in the expression of aromatase mRNA were observed, neither between control animals and 5xFAD mice nor between age groups (Figures 4(d)–4(f)). In females, however, the amount of total aromatase mRNA was significantly lower in the 3-month-old 5xFAD mice than in the controls of the same age (Figure 4(a)). In the 12-month-old 5xFAD females, expression of the ovary-specific aromatase mRNA was significantly reduced compared to the controls (Figure 4(c)). Expression of this ovary-specific transcript also changed with age in the females; in the control animals the expression increased almost 3-fold in the 12-month-old animals.

**Figure 5:**
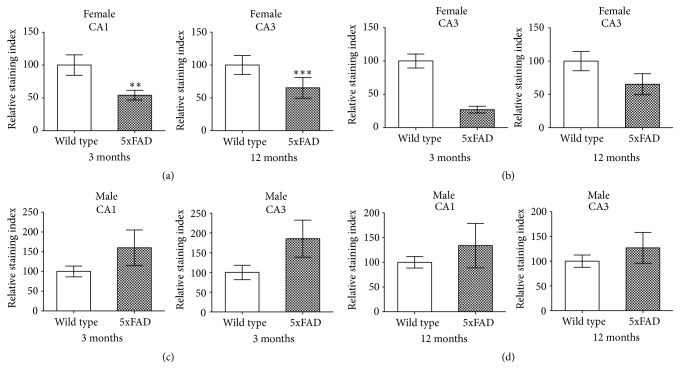
*Young female 5xFAD mice showed less aromatase immunoreactivity than WT mice*. In both hippocampal regions tested, CA1 and CA3, the relative staining index for aromatase was significantly lower in the 3-month-old female 5xFAD mice than in the WT mice (a). This difference was no longer detectable in the 12-month-old females (b). In male animals, overall the relative staining index for aromatase tended to be higher in the male 5xFAD mice; however, none of the differences was significant (c, d) (^*∗∗*^ *p* ≤ 0.01, ^*∗∗∗*^ *p* ≤ 0.001; bars represent mean ± SEM, *n* = 6).

Likewise, sex differences in the expression of aromatase protein were detected (Figure 5). In the hippocampi of male animals of both age groups, no significant differences in the relative staining index were detected in the CA1 and CA3 regions (Figures 5(c) and 5(d)). In the females, the relative staining index for aromatase was lower in the hippocampi of the 5xFAD mice with statistically significant differences in the 3-month-old mice. In the CA1 region immunostaining was reduced by 46%, in the CA3 region even by 74% (Figure 5(a)). It should be noted here that the most proximal portion of the CA3 region in rodents is comparable to the most proximal portion of the human cornu ammonis, which in the human brain is most often referred to as CA4.

## 4. Discussion

E_2_, which is synthesized by aromatase, has been shown to be neuroprotective with respect to several neurodegenerative diseases, including AD [4, 5]. It is therefore important to better understand the expression pattern of aromatase in the brain of patients with AD.

In general, results from studies using human tissue need to be interpreted with care due to high variability between samples. Here, we used samples that were matched with respect to age and sex in order to minimize variability. Although relatively long postmortem times in both groups need to be acknowledged, a recent study [29] on the quality of human tissue obtained from numerous brain banks showed that RNA quality was not affected by the extent of postmortem delays. Previously, aromatase mRNA had been detected in the human hippocampus by RT-PCR in postmortem material of male and female adults with an average age of approximately 34 years [3], and aromatase protein was detected in hippocampi obtained from a group of comparable age [30]. Our study showed that aromatase was also expressed in the hippocampi of older men and women with an average age of 77.5 years. Furthermore, the localization of aromatase within the hippocampus does not differ between younger and older people. Like Yague et al. [30], who studied a young population, we found aromatase expressed mostly in the principal neurons of the hippocampus and in a very small percentage of astrocytes in the hippocampi obtained from older people. Additionally, the subcellular localization of aromatase within the neurons observed in this study, namely, in the somata and the neurites, matches the findings in tissue samples from younger people [18, 30].

To our knowledge, this study is the first to report that neuronal aromatase expression is increased in the hippocampus of AD patients. This finding is in concordance with observations from other regions, such as the nucleus basalis of Meynert and the prefrontal cortex [31, 32]. The neuroprotective effects of the aromatase product E_2_ are well established. The hormone decreases A*β* production and increases A*β* clearance (for review [33]). Additionally, E_2_ reduces A*β*-induced cell death and A*β*-induced elevation of Ca^2+^ in hippocampal neurons [34], and it prevents the hyperphosphorylation of tau, another cause of AD (for review [35]), and has recently been shown to rescue bioenergetic deficits in mitochondria induced by A*β* and hyperphosphorylated tau [36]. It is therefore tempting to assume that an AD-related increase in aromatase expression, and therefore a potential increase in E_2_ synthesis, represents a protective reaction of the tissue to the developing disease. A recent study by Cui et al. [37] supports this idea. The authors show that the protective action of morphine against intracellular amyloid toxicity in primary neurons depends on an increase in aromatase expression and the subsequent increase in E_2_ synthesis. Furthermore, a recent study by Chang et al. [38] demonstrates that the pharmacological inhibition of aromatase with letrozole exacerbates the effects of A*β* on mitochondrial morphology and dendritic spine density in hippocampal neurons. Along those lines, we have shown earlier that treatment of organotypic hippocampal slice cultures with the aromatase substrate testosterone results in a significant increase of spine synapses [39], suggesting a potential therapeutic role of testosterone in AD. Together, these findings further stress the neuroprotective role of local aromatase and synthesis of E_2_ in AD.

Future studies will have to test what mechanisms cause the observed increase in aromatase expression in some brain regions of AD patients. It seems possible that mechanisms specifically related to the disease, such as the production or deposition of A*β*, are involved in the regulation of aromatase expression. However, it may just as well be that the tissue damage induces the upregulation of aromatase through a more general mechanism. Several experiments with rodents and birds have shown that induced, acute brain injuries result in an increased aromatase expression in hippocampal astrocytes (for review [40]). In this study of human hippocampi, aromatase immunoreactivity was only analyzed in neurons. Although some aromatase expression was observed in GFAP-positive astrocytes, obvious changes in the number of aromatase positive astrocytes or in the intensity of the aromatase staining in these cells between the AD hippocampi and the controls were not noted. This observation is in contrast with data from Luchetti et al. [32], who demonstrated an increase in aromatase mRNA expression in the prefrontal cortex of patients diagnosed with AD that coincided with an increase in the number of aromatase-immunoreactive astrocytes in this region. This increase was particularly pronounced in tissue obtained from patients at late stages of the disease. More studies focused on aromatase expression in astrocytes will be necessary to clarify whether aromatase expression is altered in astrocytes in AD-affected brains.

Furthermore, it remains unclear whether the observed increase in aromatase expression actually translates into an increase in E_2_ synthesis in the brain regions of interest. In fact, previous studies rather suggest that E_2_ levels in the brains of AD patients are lower than in control brains [12, 41]. However, as aromatase expression does not increase in all brain regions of AD, but has been shown to decrease for example in various hypothalamic nuclei in AD patients [31], it is important to take regional differences into consideration. The previously mentioned studies measured E_2_ content in the frontal cortex [41] or the frontal cortex and cerebellum [12] and may therefore not necessarily reflect the situation in the hippocampus. It may even be possible that the hippocampus itself does not show a homogeneous pattern of aromatase expression in human AD brains, as our current study is focused on aromatase expression in the CA4 region. Previously, we have demonstrated that aromatase is differentially expressed in the CA1 and CA3 region of rat hippocampi [42]. Given that previous experiments suggest that hippocampus-derived E_2_ acts in a paracrine and/or autocrine fashion [23, 42], the analysis of steroid concentrations within individual regions of the human hippocampus would be particularly valuable.

The circumstance that aromatase is upregulated in some regions of AD brains (e.g., the CA4 region of the hippocampus (shown in this study), the prefrontal cortex [32], and the nucleus basalis of Meynert [31]), but downregulated in other regions (e.g., the supraoptic nucleus, the infundibular nucleus, and the medial mamillary nucleus [31]), suggests that the regulation of aromatase expression is very complex. Several first exons and their respective promoters, which were initially described as tissue-specific [15], play an important role in this regulation. Initially, the I.f promoter was identified as the brain-specific aromatase promoter [16]. In subsequent studies, other first exons, namely, PII, I.4, I.3, and a truncated form of I.3 (I.3T), have been detected, along with I.f in various regions of the human brain [17, 18]. Our findings differ from the previous findings, as we did not detect the I.4 first exon, which was prominent in some of the human hippocampi test by Sasano et al. [17]; instead, we detected the H2 variant of exon I.6. In the study by Sasano et al. [17], the expression pattern of the first exons did not only vary between regions but also between the tested individuals. The detection of additional first exons in the hippocampi is therefore not really surprising; it rather stresses further the variability in aromatase regulation. In breast tissue, it has been shown that the use of alternate first exons changes not only with age but also with malignant transformation [43, 44]. Interestingly, the alternative first exons have also been shown to differentially modulate aromatase gene expression via posttranscriptional regulation [45]. In the long term, learning more about potential changes in the use of tissue-specific first exons of aromatase in neurological diseases may open pathways to novel treatment options because the promoters have different regulatory elements, which could be used for a selective regulation [15]. It should also be emphasized that the data shown here reflect the first exons present in the entire hippocampus. Additional experiments using tissue from defined regions (e.g., CA1, CA3, and CA4) would be necessary to make statements about aromatase mRNA expression patterns within the various regions of the hippocampus.

The complexity of aromatase regulation also becomes obvious when studying the 5xFAD mouse model for AD. Most notably, sex differences in the regulation of aromatase expression were observed. While no significant differences in the expression of aromatase mRNA or protein were detected in the male groups, in the females, the expression pattern varied between the 5xFAD mice and the control mice. In humans, sex differences in the development of AD are frequently discussed; for example, the prevalence of AD as well as the rate of decline is higher in women than in men (for review [14, 46]). In general, the strong decline of circulating E_2_ due to menopause is assumed to be a major risk factor for AD in women [35, 47]. Recent studies in an AD animal model that also expresses less aromatase (APP23/Aro^+/−^ mice), however, indicate that aromatase expression in the brain may account for some of the observed sex differences. While female APP23/Aro^+/−^ mice developed more A*β* plaques at an earlier age than ovariectomized APP mice, which still could synthesize E_2_ in the brain [12], male APP23/Aro^+/−^ mice showed a reduced rate of plaque deposition compared to APP23 control mice [13]. This indicates that the function of brain-derived aromatase differs between male and female animals.

In the female mice, we observed a significant decrease in the amount of total aromatase mRNA expression in the 3-month-old animals, which was accompanied by less aromatase protein expression in the CA1 and CA3 regions. It is possible that the increased production of A*β* inhibits aromatase expression in the young mice, which in turn results in the loss of neuroprotection by E_2_. Interestingly, a study using the APP23/Aro^+/−^ mouse model recently showed that in these mice only early treatment with E_2_ (starting at three months of age) resulted in less plaque formation, whereas later treatment (starting at nine months of age) did no longer prevent or reverse plaque formation [48]. These data, together with our current findings, suggest that E_2_ can only be neuroprotective if sufficient E_2_ levels are met in the brain before morphological changes, such as A*β* plaque formation, occur. This assumption is in line with the idea of a “window of opportunity” (for review [14]): during a defined time interval, E_2_ treatment could prevent or at least slow down the development of AD in women. Once the brain has been deprived of E_2_ for an extended period of time, this effect no longer occurs and hormone treatment might even be detrimental to cognition.

In the hippocampus of the female 5xFAD mice, the various first exons were regulated differently: hardly any changes were observed in the expression of the brain-specific first exon, whereas the ovary-specific first exon was highly expressed in the 12-month-old WT animals. Surprisingly, this differential regulation was not reflected in the amount of total aromatase mRNA, which was not significantly higher in the 12-month-old WT mice compared to the 5xFAD mice and the 3-month-old WT mice. The discrepancy between the results on total aromatase mRNA and on ovary-specific first exon mRNA could be explained by the existence of other, as yet undescribed, variants of aromatase, which are regulated opposite to the ovary-specific first exon and, therefore, would balance the amount of total aromatase. Alternatively, the ovary-specific variant of aromatase could exist in a truncated form, which does not contain exons 8 and 9, and is therefore undetected by the primers used to quantify total aromatase. Truncated aromatase mRNA has been found in other tissues, for example, in the granulosa cells of rabbits and in the testicular cells of rats [49–51].

The expression pattern of total aromatase mRNA in the hippocampus seems to coincide with the pattern of aromatase immunoreactivity in CA1 and CA3. However, one should keep in mind that the entire hippocampus was used for real-time PCR analysis. Therefore, conclusions on the regional expression patterns of the mRNA are not possible.

Unlike in the human hippocampi, we did not observe higher aromatase immunoreactivity in the hippocampi of the older 5xFAD mice than in the control mice. However, the difference that was observed in the younger females (lower expression in 5xFAD) did not exist in the older animals, suggesting that aromatase expression increased in the older 5xFAD mice.

Our studies indicate that hippocampal aromatase expression can be effected by AD. However, more research will be necessary to explain the observed differences in aromatase expression in humans with AD and in 5xFAD mice and to clarify whether this mouse model is suitable to investigate steroid hormone-mediated neuroprotection in this disease.

## 5. Conclusion

Our study suggests that hippocampal aromatase expression may change in response to Alzheimer's disease in both humans and mice and supports the idea that brain-derived E_2_ has neuroprotective function. Furthermore, the studies on mouse tissue emphasize the importance of hippocampal aromatase expression, and therefore E_2_ synthesis, in the early stages of AD. The sex differences found in the mouse model are in agreement with previous findings, which indicate that the neuroprotective role of brain-derived estradiol may be more important in females than in males [12, 13] and may help to explain why women are more prone to the disease than men [14].

## Supplementary Material

Table S1 provides detailed information (e.g. sex, age, postmortem time, etc.) about the human tissue samples that were used in this study. Sample pairs 1-10 were used for immunohistochemical analysis; samples 11-18 were used for PCR analysis.
Table S2 lists the sequences of the first exon-specific primers that were used to detect various variants of aromatase mRNA in human tissue. The table also provides the specific annealing temperatures for each primer pair and the length of the resulting PCR product.
Table S3 lists the sequences of the first exon-specific primers that were used to detect various variants of aromatase mRNA in mouse tissue. The table also provides the specific annealing temperatures for each primer pair and the length of the resulting PCR product.
Figure S1 Co-immunostaining for the astrocyte marker glial fibrillary acidic protein (GFAP) and aromatase revealed that most astrocytes in the human hippocampus did express aromatase at very low levels or not at all.
