# Supplementary material for: Aromatase Expression in the Hippocampus of AD Patients and 5xFAD Mice
Source: Neural Plast. 2016 May 19;2016:9802086. doi: 10.1155/2016/9802086 (PMC4889864; doi:10.1155/2016/9802086)
Supplement: Supplementary file 1 — Table S1 provides detailed information (e.g. sex, age, postmortem time, etc.) about the human tissue samples that were used in this study. Sample pairs 1-10 were used for immunohistochemical analysis; samples 11-18 were used for PCR analysis. Table S2 lists the sequences of the first exon-specific primers that were used to detect various variants of aromatase mRNA in human tissue. The table also provides the specific annealing temperatures for each primer pair and the length of the resulting PCR product. Table S3 lists the sequences of the first exon-specific primers that were used to detect various variants of aromatase mRNA in mouse tissue. The table also provides the specific annealing temperatures for each primer pair and the length of the resulting PCR product. Figure S1 Co-immunostaining for the astrocyte marker glial fibrillary acidic protein (GFAP) and aromatase revealed that most astrocytes in the human hippocampus did express aromatase at very low levels or not at all. [file 9802086.f1.pdf]

Table S1: Data on human tissue samples

#	Controls						Patients diagnosed with AD						
	**Internal processing number**	**Gender**	**Age [y]**	**Year of death**	**Post-mortem time [h]**	**Pro-cess-ing***	**Internal processing number**	**Gender**	**Age [y]**	**Year of death**	**Braak**	**Post-mortem time [h]**	**Pro-cess-ing***
**1**	NP31	f	54	2006	168	Paraf.	344	f	54	1998	6	NA	Paraf.
**2**	28	m	58	2006	144	Paraf.	61	m	56	2006	2	120	Paraf.
**3**	56	m	69	2006	48	Paraf.	189	m	73	2000	2	77	Paraf.
**4**	NP21	f	79	2006	120	Paraf.	NP7	f	79	1999	5	72	Paraf.
**5**	149	f	82	2005	NA	Paraf.	203	f	80	1998	4	48	Paraf.
**6**	70	f	82	2006	264	Paraf.	17	f	81	2006	3	192	Paraf.
**7**	19	f	84	2005	48	Paraf.	103	f	86	1999	4	96	Paraf.
**8**	62	f	84	2006	48	Paraf.	9	f	87	1995	NA	NA	Paraf.
**9**	NP90	f	89	2005	144	Paraf.	NP120	f	89	2000	6	24	Paraf.
**10**	38	f	91	2006	144	Paraf.	65	f	92	2000	6	48	Paraf.
**11**	83	f	69	2008	72	Frozen							
**12**	224	m	87	2008	96	Frozen							
**13**	246	m	61	2008	96	Frozen							
**14**	368	m	68	2008	120	Frozen							
**15**							202	m	74	2007	1	48	Frozen
**16**							7	f	80	2008	3	120	Frozen
**17**							168	m	81	2008	3	48	Frozen
**18**							244	m	83	2008	5	120	Frozen

* Parafin-embedded (Paraf.) samples were used for immunohistochemistry; snap-frozen (Frozen) samples were used for RT-PC

Table S2: First exon-specific aromatase primers for human tissue

Name	Sequence	Annealing Temp.	Length
Exon PII	f: 5'-GCA ACA GGA GCT ATA GAT GAA C-3'	63 ºC	236 bp
	r: 5'-GTG CCC TCA TAA TTC CAC AC-3'		
Exon I.f	f: 5'-GAA AAG CCA CCT GGT TCT TA-3'	63 ºC	261 bp
	r: 5'-GTG CCC TCA TAA TTC CAC AC-3'		
Exon I.3	f: 5'-AGG AAC CTG AGA CTC TAC CAA G-3'	63 ºC	207 bp
	r: 5'-GTG CCC TCA TAA TTC CAC AC-3'		
Exon I.4	f: 5'-TCC TGG CTC CAA GTA GAA CGT GAC-3'	69 ºC	118 bp
	r: 5'-ACC ATC TTG TGT TCC TTG ACC TCA G-3'		
Exon I.6	f: 5'-AGG GAT TAC AAA ACC TGG CTG AA-3'	69 ºC	897 bp
	r: 5'-ACC ATC TTG TGT TCC TTG ACC TCA G-3'		

Table S3: Primers for mouse tissue

Name	Sequence	Annealing Temp.	Length
Aro_total	f: 5’-GAG GTC GAA GCA GCA ATC CT-3’<br>r: 5’-GAT ATC CTC GAT CTT TAT GTC TCT GTC A-3’	60 ºC	72 bp
Aro_ovary	f: 5’-ACA AAA TGC CCA TCT CTC CAA-3’<br>r: 5’-GTG CCC TCA TAA TTC CAC AC-3’	60 ºC	57 bp
Aro_brain	f: 5’-AAC TCA CCA TCT TCA AGA GTC CA-3’<br>r: 5’-GAG TGG CAT GGC ACT GAC AGT-3’	60 ºC	149 bp
Actin-beta	f: 5-CTG GCT CCT AGC ACC ATG AAG’3’<br>r: 5’-CAC CGA TCC ACA CAG AGT ACT TG-3’	60 ºC	70 bp

Legends

Figure S1: *Hippocampal astrocytes showed very low levels of aromatase expression.* Co-immunostaining (C) for the astrocyte marker glial fibrillary acidic protein (GFAP, A) and aromatase (B) revealed that most astrocytes in the human hippocampus did express aromatase at very low levels or not at all. Nuclei were stained blue with DAPI. Scale bar: 50 μm.

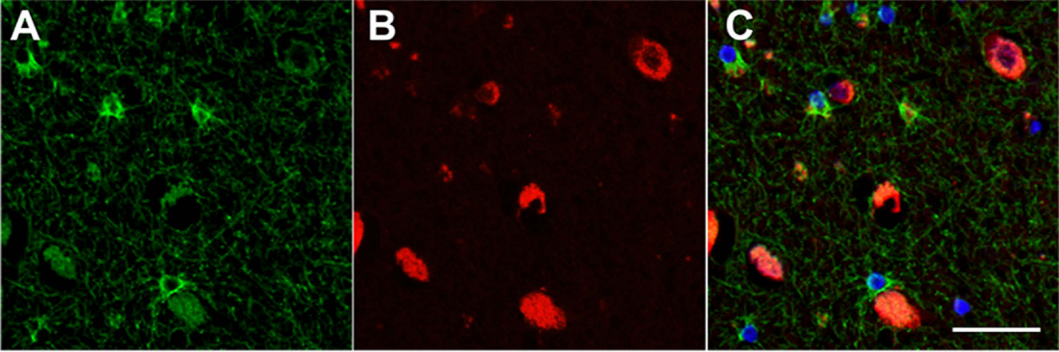

Fig. S1
